# Health misinformation, digital health literacy, and attitudes toward science among university students: an exploratory cross-sectional survey

**DOI:** 10.3389/fpubh.2026.1931903

**Published:** 2026-08-31

**Authors:** Adele Sarcone, Claudia Pileggi, Carmelo Nobile, Silvio Simeone, Caterina Mercuri

**Affiliations:** 1 Health Sciences Department, Magna Graecia University, Catanzaro, Italy; 2 Clinical and Experimental Medicine Department, Magna Graecia University, Catanzaro, Italy; 3 Department of Pharmacy, Health and Nutritional Sciences, University of Calabria, Cosenza, Italy

**Keywords:** digital health literacy, fake news, health misinformation, infodemic, social media

## Abstract

**Background:**

The spread of health misinformation through digital platforms poses an increasing challenge for public health. University students are among the most exposed groups, given their heavy reliance on online environments for health-related information.

**Objective:**

This study investigated health information sources, digital health literacy skills, criteria for evaluating information quality, use of homeopathic and alternative remedies, and attitudes toward science among students at the University Magna Graecia of Catanzaro (UMG), Italy.

**Methods:**

An exploratory cross-sectional survey was conducted on 407 UMG students (277 females, 68.06%; mean age 23.23 ± 6.15 years), enrolled in Nursing (65.36%), Medicine and Surgery (23.34%), Engineering (2.46%), and other programmes (8.85%). Data were collected via a structured self-administered questionnaire distributed through Google Forms. Descriptive statistics, independent-samples *t*-tests, chi-square tests, and stepwise logistic regression were performed.

**Results:**

Social media was the most frequently used health information source (75.43%), yet only 12.78% of students considered it reliable. The physician was identified as the most trustworthy source by 94.10% of participants. Basic digital health behaviors were common, but advanced self-reported critical evaluation abilities were markedly limited: only 15.72% self-reported the ability to critically select online health content. Attitudes toward science were generally positive. Chi-square analyses revealed sex differences in response distributions for several items, and logistic regression showed that female sex was positively associated with sensitivity to the ethical dimension of scientific work (OR = 1.61).

**Conclusions:**

Positive scientific attitudes coexisted with underdeveloped self-reported critical evaluation abilities. These findings support the integration of digital health literacy training into university curricula to better prepare students to navigate the contemporary infodemic environment.

## 1 Introduction

The uncontrolled dissemination of false or misleading health information via digital channels represents one of the most complex and urgent challenges currently facing Public Health (1, 2). The rapid expansion of social media platforms and the progressive disintermediation of scientific communication have profoundly altered the manner in which citizens access, select, and interpret health-related content, generating an information ecosystem in which the epistemic quality of content is no longer guaranteed by the institutional origin of the source, but depends increasingly on the critical competencies of the individual user (3–5).

Health misinformation, the circulation of false, inaccurate, or misleading health-related information regardless of intent, as emerged as a structural threat to global Public Health, with documented effects on preventive, diagnostic, and therapeutic decision-making at both individual and population levels (3, 6). The COVID-19 pandemic epitomized this challenge through the concept of the “infodemic,” coined by the WHO to describe the overabundance of information accompanying the virus's spread, with tangible consequences for preventive behaviors, vaccine uptake, and institutional trust (6–9).

The consequences of health misinformation are not confined to the pandemic context; rather, they persistently permeate the entire continuum of prevention, diagnosis, and treatment (10). Vaccine hesitancy, recourse to therapies lacking scientific evidence, reduced adherence to clinical recommendations, and the proliferation of conspiratorial narratives that erode trust in the scientific community and health institutions represent well-documented manifestations of a phenomenon that operates continuously and pervasively on individual and collective health-related decision-making (11–14). Jabbour et al. (15) found that prolonged exposure to specific social media platforms significantly undermines favorable vaccination attitudes, while Fendt et al. (16) demonstrated that university students, despite awareness of misinformation, lack systematic critical tools and rely predominantly on intuitive heuristics to evaluate health content.

The university population occupies a position of analytical and strategic relevance in this scenario.

Social media represent the preferred information channel among university students, who access them daily and tend to share health-related content without prior critical verification (7, 17, 18). Studies document that the majority of students receive health news via social media and, while attempting cross-referencing with other sources, rarely adopt systematic verification strategies, thereby amplifying misinformation spread (7, 19, 20).

This tension between intensive use of digital platforms and limited critical awareness of their shortcomings represents one of the central challenges of contemporary health education, documented consistently across diverse geographical and cultural contexts (21–24).

Within this framework, the concept of digital health literacy, defined as the ability to locate, understand, critically evaluate, and apply health information retrieved through digital sources, emerges as a fundamental competency not only for future health professionals, but for all citizens navigating the contemporary information ecosystem (25–27).

Structured educational interventions have proven effective in improving digital health literacy among medical students (28), while sex-related differences have also been documented, with female students showing greater tendency to verify source reliability before making health decisions (17).

The university setting therefore constitutes a privileged context for the integrated investigation of these phenomena, bringing together young, digitally active populations in the process of constructing their epistemic and professional identities. In particular, students enrolled in health-related degree programmes, such as Nursing and Medicine and Surgery, are destined to serve as qualified mediators between scientific knowledge and the general population, rendering their critical training with respect to misinformation not merely an individual educational objective, but a systemic priority with direct implications for the quality of care and the promotion of Public Health (29–32). The contemporary university, however, accommodates increasingly heterogeneous student populations: alongside students enrolled in health programmes, those from technical and scientific disciplines, such as Engineering, share the same digital environments, the same information platforms, and the same epistemological challenges, thereby rendering digital health literacy a transversal competency that cannot be confined exclusively to health curricula. The inclusion of non-health discipline students, although proportionally limited, was therefore deliberate: it enables exploratory discipline-based comparisons that may either confirm the transversal nature of digital health literacy deficits across the university population or reveal discipline-specific patterns with implications for curriculum design. With regard to complementary and alternative medicine, international studies conducted among medical and nursing students have documented the persistence of favorable attitudes toward such practices even within educational contexts oriented toward evidence-based practice, underscoring the importance of understanding the attitudes of future health professionals in order to design interventions capable of reinforcing adherence to the principles of evidence-based medicine (33–35). Despite the growing body of international scientific literature on these topics, relevant knowledge gaps remain. Existing studies have predominantly examined health misinformation, digital health literacy, and attitudes toward science as separate constructs rather than as components of an integrated investigative framework. Italian evidence, in particular, remains limited in scope: available studies have largely targeted homogeneous samples drawn exclusively from health disciplines, derive predominantly from universities in northern and central Italy, and have rarely explored how these dimensions vary according to sex and year of study. This fragmentation constrains the identification of priority targets for evidence-based educational interventions. The present study aimed primarily to provide a descriptive overview of health information sources, digital health literacy skills, criteria used to evaluate online health information, attitudes toward science, and the use of alternative remedies among students at the University Magna Graecia of Catanzaro (UMG), a university in southern Italy enrolling students across Nursing, Medicine and Surgery, Engineering, and other degree programmes. In addition, the study explored potential differences according to sex and year of study across selected dimensions related to trust in science, misinformation, and epistemological attitudes.

The findings are intended to provide an empirical basis for the design of targeted educational interventions aimed at strengthening digital health literacy and critical evaluation skills among future professionals navigating the contemporary infodemic environment. The study was conceived primarily as a descriptive and exploratory investigation aimed at generating hypotheses rather than formally testing prespecified hypotheses or causal relationships.

## 2 Materials and methods

### 2.1 Study design and setting

An exploratory cross-sectional survey was conducted to assess the modalities of access to and critical evaluation of health information, the perception of misinformation in the scientific domain, and university students' attitudes toward science and the scientific method. The study was carried out at the University Magna Graecia of Catanzaro (UMG), Catanzaro, Italy.

### 2.2 Study population

The study population comprised university students aged ≥ 18 years, regularly enrolled in bachelor's, master's, or single-cycle degree programmes at the University Magna Graecia of Catanzaro (UMG). Participants were enrolled in various degree programmes, including Nursing, Medicine and Surgery, Engineering, and others. Students were eligible irrespective of their year of enrolment or prior exposure to educational programmes in scientific communication or media literacy.

Data collection was conducted between September 2025 and January 2026, during scheduled teaching activities involving students.

### 2.3 Inclusion and exclusion criteria

Inclusion criteria:

- Age ≥ 18 years;
- Active enrolment in a degree programme at UMG;
- Voluntary completion of the questionnaire;
- Provision of informed consent.

Exclusion criteria:

- Age < 18 years;
- Incomplete or invalid questionnaires;
- Refusal to provide informed consent.

### 2.4 Sample size and recruitment

Participants were recruited using a convenience sampling strategy, through the administration of a structured questionnaire during face-to-face teaching sessions. Participation was voluntary and anonymous. The resulting sample composition, characterized by a predominance of students enrolled in health-related degree programmes, is consistent with the actual distribution of student enrolment across the degree programmes of the University Magna Graecia of Catanzaro, where health disciplines represent the majority of the academic offer. Given the exploratory nature of the study, no *a priori* power calculation was conducted. The sample size was determined by the number of students attending the scheduled teaching sessions during the data collection period.

It should be noted that 71.50% of participants were enrolled in the first year of study; this distribution reflects the recruitment context and should be taken into account when interpreting the stratified analyses by academic year.

### 2.5 Data collection instrument

Data were collected by means of a self-administered structured questionnaire developed *ad hoc* by the research team informed by literature, drawing on the available scientific literature on health literacy, the infodemic, and the public perception of science, and entitled “Fake News and Public Health.” The questionnaire was administered via the Google Forms platform (Google LLC, Mountain View, CA, USA), enabling secure, anonymous, and standardized data collection. Prior to final administration, the instrument was subjected to expert review, with expertise in public health and health education to assess the clarity and relevance of its content. No formal psychometric validation procedures were conducted (internal consistency analysis, exploratory factor analysis, or test-retest reliability assessment). The instrument was therefore used for exploratory purposes only.

The questionnaire was divided into four main sections:

Section A in Supplementary material, Sociodemographic and Academic Characteristics. This section collected information on age (A1), gender (A2: male, female), marital status (A3), degree programme (A4), and year of study (A5).

Section B in Supplementary material, Access to and Quality of Health Information. This section investigated: (i) the channels habitually used to access information on Public Health and science (B1, multiple response); (ii) the ability to access and navigate online health information as an indicator of *digital health literacy* (B2, multiple response, six options); and (iii) the criteria adopted to evaluate the quality of health information (B3, multiple response).

Section C in Supplementary material, Perception of Science and Information Sources. This section explored: (i) the health information source considered most reliable (C.2); and (ii) the perceived probability that the scientific environment may be influenced by external third-party entities (C.3). Items relating to the use of homeopathic products (B.4) and the propensity to recommend alternative remedies (C.4) were also included in this domain.

Section D in Supplementary material, Attitudes toward Science. This section comprised a five-point Likert scale (0 = “Strongly disagree” to 4 = “Strongly agree”) consisting of 14 items, developed *ad hoc* by the research team, drawing on the available scientific literature on trust in science and scientists (16, 17), and adapted to the Italian context and the target population of university students.

Prior to analysis, all submissions were screened for duplicate entries; no duplicates were identified. Items with missing responses were excluded from the relevant analyses on a pairwise basis; the overall rate of missing data was negligible.

### 2.6 Study variables

The main study variables included sociodemographic characteristics (age, sex, degree programme, year of study), variables related to health information-seeking behaviors and digital health literacy, attitudes toward science measured through the 14-item Likert scale (D1–D14), trust in information sources, and the use of homeopathic or alternative remedies.

### 2.7 Statistical analysis

Statistical analyses were performed using Stata software (StataCorp LLC, College Station, TX, USA). Continuous variables were summarized as means and standard deviations (SD), whereas categorical variables were reported as absolute frequencies and percentages.

Sex differences in attitudes toward science were explored using two-sided independent-samples *t*-tests for mean item scores and chi-square tests for response distributions, with statistical significance set at *p* < 0.05. No correction for multiple comparisons was applied, consistent with the exploratory nature of the study. All reported *p*-values should therefore be interpreted with appropriate caution, particularly in light of the number of statistical tests performed. To explore the association between sex and selected variables related to attitudes toward science and trust in information sources, a stepwise logistic regression model with backward elimination was performed, using sex as the binary dependent variable (0 = male, 1 = female). Odds ratios (ORs) and 95% confidence intervals (95% CIs) were reported. The backward elimination procedure was selected in the absence of a pre-specified theoretical model for variable selection, in keeping with the exploratory intent of the analysis. This approach was considered appropriate for generating preliminary hypotheses regarding sex-differentiated attitudinal profiles in an under-investigated population; it was not intended to support confirmatory inference. The recognized limitations of stepwise procedures, including potential model instability and the risk of capitalizing on chance associations, are explicitly acknowledged among the study limitations. The model was specified to address a targeted research question,namely the identification of attitudinal, behavioral, and sociodemographic variables most strongly associated with biological sex in this sample. Positioning group membership as the criterion variable is methodologically consistent with discriminant function analysis (DFA) and is the recommended approach when distributional assumptions on the predictors cannot be guaranteed (36, 37). This approach is supported by peer-reviewed health research in which logistic regression with biological sex as the dependent variable is employed to identify variables that differ systematically between male and female participants (38–40). The resulting odds ratios are interpreted as descriptive measures of differential association rather than causal estimates (41). For the descriptive analysis stratified by year of study, frequency distributions of the main study variables were calculated within each academic year. No formal inferential comparisons between year groups were performed, owing to the marked imbalance in subgroup sizes and the limited number of participants in several strata. While alternative modeling strategies, notably, modeling attitudinal variables as dependent outcomes, may have offered a more conventional epidemiological framing, the present analysis was designed as exploratory from the outset; the modeling approach was selected accordingly and is explicitly recognized as a limitation of the study. Stratified analyses by academic year were performed for descriptive purposes; given the unequal distribution across cohorts, between-group comparisons should be interpreted with caution.

### 2.8 Ethical considerations

The study was conducted in accordance with the principles of the Declaration of Helsinki and applicable Italian legislation on the protection of personal data (Legislative Decree No. 196/2003 and subsequent amendments; EU Regulation 2016/679, GDPR). The study protocol was approved by the Ethics Committee of the Calabria Region (protocol n 363, date 20 December 2024). Participation was voluntary and anonymous; all participants provided written informed consent prior to completing the questionnaire. Data were processed in anonymised and aggregated form, ensuring the confidentiality of participants at every stage of the research process.

## 3 Results

### 3.1 Sociodemographic characteristics of the sample

The final sample comprised 407 university students enrolled at the University Magna Graecia of Catanzaro. The mean age of participants was 23.23 years (SD = 6.15; range: 18–55 years). The sample was predominantly female (*n* = 277; 68.06%), with males representing 31.94% (*n* = 130). With respect to marital status, the vast majority of participants were single or never married (*n* = 360; 88.45%), followed by those who were married or cohabiting (*n* = 42; 10.32%), separated or divorced (*n* = 4; 0.98%), and widowed (*n* = 1; 0.25%).

**Figure 1:**
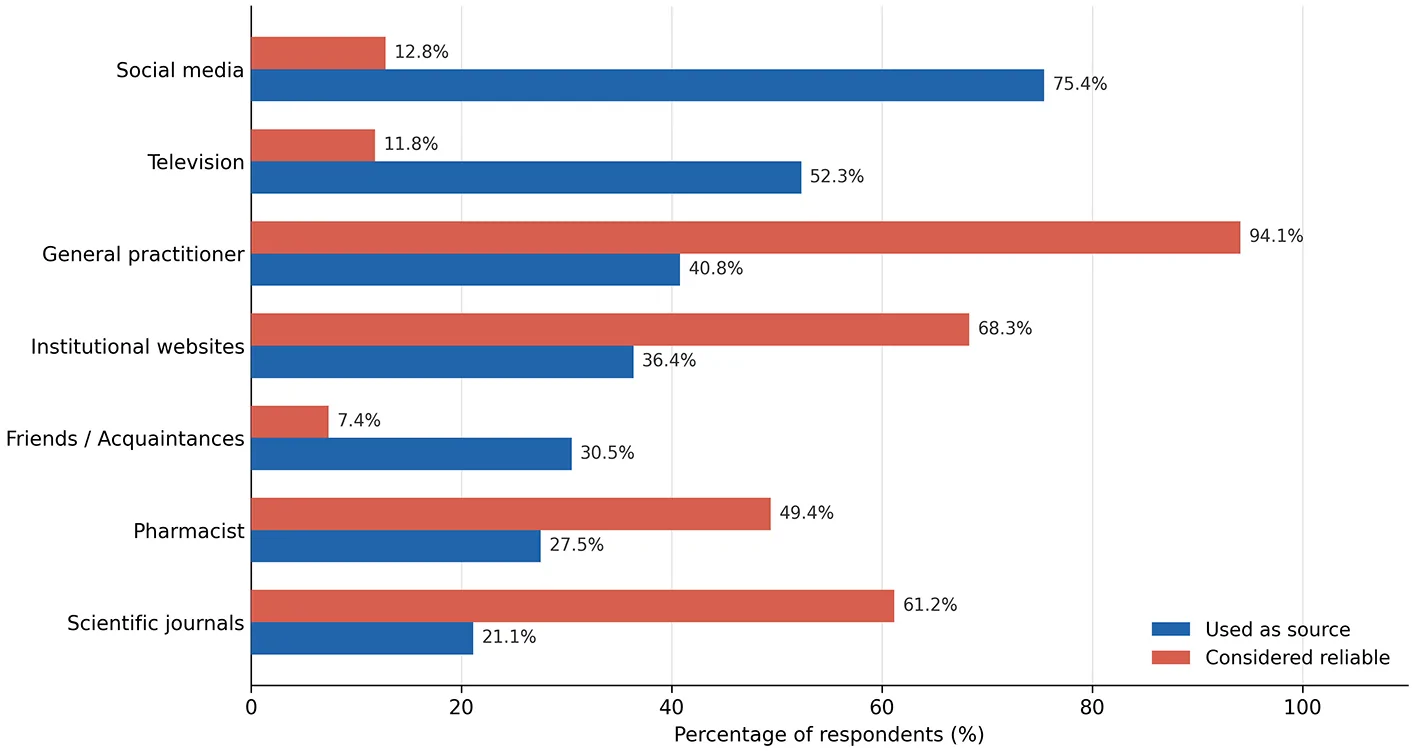
Health information sources: used vs. considered reliable (*N* = 407; multiple-response item).

Regarding degree programme, the distribution demonstrated a clear prevalence of students enrolled in Nursing (*n* = 266; 65.36%), followed by Medicine and Surgery (*n* = 95; 23.34%), other programmes (*n* = 36; 8.85%), and Engineering (*n* = 10; 2.46%). The distribution by year of study demonstrated a marked concentration in the first year (*n* = 291; 71.50%), with progressively smaller proportions in subsequent years: fourth year (*n* = 36; 8.85%), sixth year (*n* = 28; 6.88%), fifth year (*n* = 16; 3.93%), third year (*n* = 15; 3.69%), second year (*n* = 14; 3.44%), and out-of-programme students (*n* = 7; 1.72%). Complete sociodemographic data are reported in Table 1, and the distribution by degree programme is illustrated in Figure 1.

**Table 1:** Sociodemographic characteristics of the sample (*N* = 407).

Characteristic	*n*	%
Sex		
Female	277	68.06
Male	130	31.94
Age (years)		
Mean ± SD	23.23 ± 6.15	n/a
Range	18–55	n/a
Marital status		
Single/never married	360	88.45
Married/cohabiting	42	10.32
Separated/divorced	4	0.98
Widowed	1	0.25
Degree programme		
Nursing	266	65.36
Medicine and surgery	95	23.34
Other programmes	36	8.85
Engineering	10	2.46
Year of study		
1st year	291	71.50
2nd year	14	3.44
3rd year	15	3.69
4th year	36	8.85
5th year	16	3.93
6th year	28	6.88
Out-of-programme	7	1.72

### 3.2 Health information sources

Analysis of health information sources (multiple-response item) revealed that social media constituted the most frequently used channel (*n* = 307; 75.43%), followed by television (*n* = 213; 52.33%), the general practitioner (*n* = 166; 40.79%), and friends or acquaintances (*n* = 124; 30.47%). Only 0.74% of participants (*n* = 3) reported that public health and science information was not of interest to them. Complete data are reported in Table 2; the comparison between used and reliable sources is illustrated in Figure 1.

**Table 2:** Health information sources used (multiple response; *N* = 407).

Information source	*N*	%
Social media	307	75.43
Television	213	52.33
General practitioner	166	40.79
Friends/acquaintances	124	30.47
Not of interest	3	0.74

### 3.3 Digital health literacy skills

**Figure 2:**
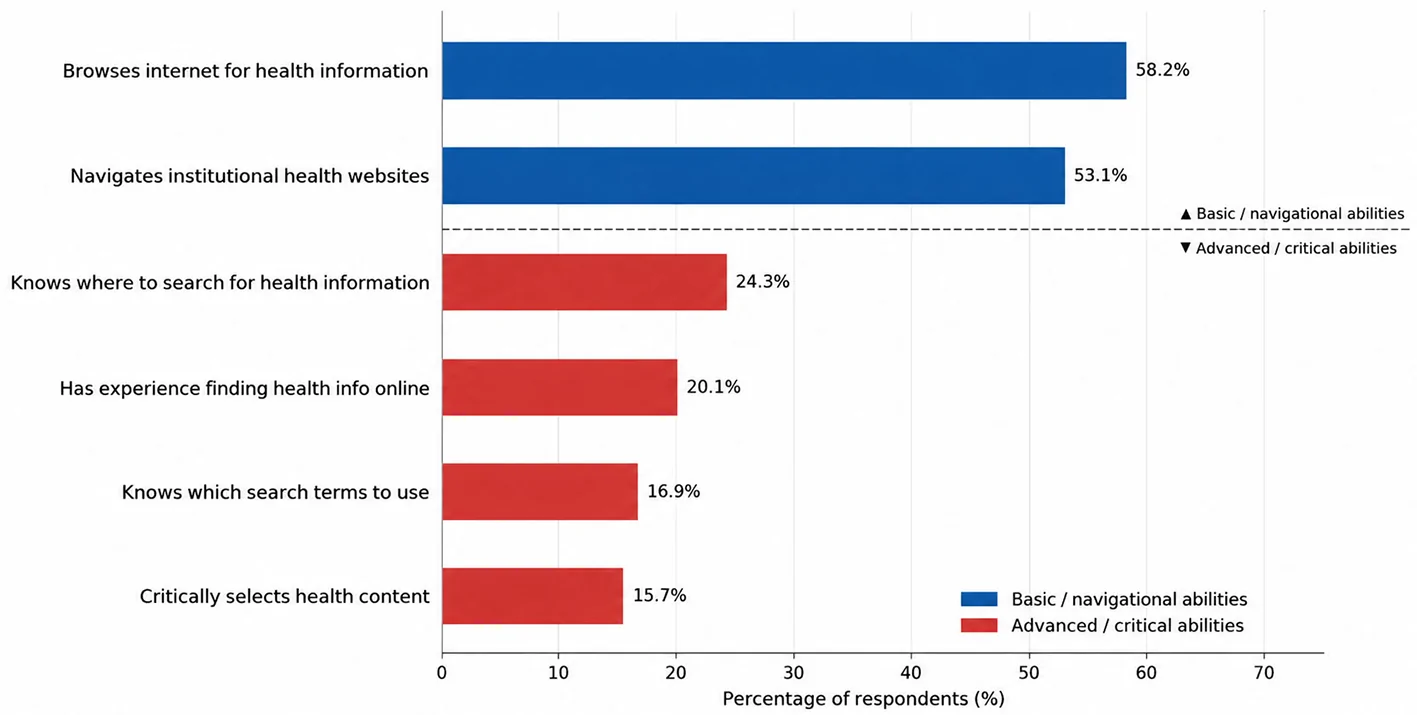
Self-reported digital health literacy abilities (*N* = 407; multiple-response item).

Analysis of declared digital health literacy skills (multiple-response item; B.2) revealed a profile characterized by a predominance of basic navigational behaviors and a marked underrepresentation of self-reported critical evaluation abilities. The most frequently reported behavior was browsing the internet for further health-related information upon encountering health content (*n* = 237; 58.23%), followed by navigating public health institutional websites or hospital portals when seeking health information (*n* = 216; 53.07%). Intermediate competencies were considerably less prevalent: only 24.32% (*n* = 99) reported knowing where to search for health information online, and 20.15% (*n* = 82) declared having experience in finding health-related information on the internet. Advanced search skills were reported by a minority of students: 16.95% (*n* = 69) stated they know which search terms to use, and only 15.72% (*n* = 64) reported being able to critically select the desired information from the vast pool of health content available online. Complete data are reported in Table 3; the distribuition of self-reported abilities levels is illustrated in Figure 2.

**Table 3:** Self-reported digital health literacy abilities (multiple response; *N* = 407).

Skill	*n*	% of cases
Browses the internet for further health-related information	237	58.23
Navigates public health institutional websites or hospital portals	216	53.07
Knows where to search for health information online	99	24.32
Has experience finding health-related information online	82	20.15
Knows which search terms to use for health information	69	16.95
Can critically select desired information from available health content	64	15.72

### 3.4 Criteria for evaluating information quality

Analysis of the criteria adopted to evaluate the quality of health information (multiple-response item B.3; nine categories) revealed that the most frequently cited criterion was up-to-date content (*n* = 200; 49.14%), followed by information provided by health professionals (*n* = 184; 45.21%), source verification (*n* = 168; 41.28%), and accuracy of information (*n* = 138; 33.91%). Scientific evidence was cited by 32.43% (*n* = 132) of participants. Less frequently adopted criteria included cross-checking with multiple websites (*n* = 107; 26.29%), reporting only positive effects or concealing risks (*n* = 80; 19.66%), consultation with a health professional (*n* = 67; 16.46%), and transparency of intentions/purposes (*n* = 59; 14.50%). Complete data are reported in Table 4.

**Table 4:** Criteria for evaluating health information quality (multiple response; *N* = 407).

Criterion	*N*	% of cases
Up-to-date content	200	49.14
Information provided by health professionals	184	45.21
Source verification	168	41.28
Accuracy of information	138	33.91
Scientific evidence	132	32.43
Cross-checking with multiple websites	107	26.29
Reporting only positive effects or concealing risks	80	19.66
Consultation with a health professional	67	16.46
Transparency of intentions/purposes	59	14.50

### 3.5 Use of homeopathy and alternative remedies

Analysis of homeopathic product use revealed that 21.13% of participants (*n* = 86) reported using such products. Prevalence was slightly higher among females (23.10%; *n* = 64/277) than among males (16.92%; *n* = 22/130). Regarding the propensity to recommend alternative remedies, only 9.09% of participants (*n* = 37) expressed a willingness to do so. Complete data are reported in Table 5.

**Table 5:** Use of homeopathy and propensity to recommend alternative remedies by sex (*N* = 407).

Variable	Total (*N* = 407)	Females (*n* = 277)	Males (*n* = 130)
Use of homeopathy (Yes, %)	21.13	23.10	16.92
Recommends alternative remedies (Yes, %)	9.09	9.39	8.46

### 3.6 Trust in information sources and the role of scientists

Analysis of the health information source considered most reliable revealed near-unanimous trust in the physician (*n* = 383; 94.10%), which substantially outranked all other sources. Politicians were indicated by 0.25% (*n* = 1), religious authorities by 0.49% (*n* = 2), citizen groups by 0.98% (*n* = 4), friends and family by 1.97% (*n* = 8), journalists by 0.98% (*n* = 4), and NGOs by 1.23% (*n* = 5).

Regarding the perceived probability that the scientific environment may be influenced by external third-party entities, 46.68% of participants (*n* = 190) considered this “probable”, 33.66% (*n* = 137) “possible,” 12.29% (*n* = 50) “certain,” 6.14% (*n* = 25) “unlikely,” and 1.23% (*n* = 5) “impossible.” Data are reported in Table 6.

**Table 6:** Perceived probability of external influence on the scientific environment (*N* = 407).

Response	*n*	%
Probable	190	46.68
Possible	137	33.66
Certain	50	12.29
Unlikely	25	6.14
Impossible	5	1.23

### 3.7 Attitudes toward science: analysis of the likert scale

Analysis of attitudes toward science using the 14-item Likert scale revealed an overall positive profile. Items reflecting trust in scientific work obtained the highest mean scores among the positively worded items: “We should trust the work of scientists” (D.9; *M* = 2.95; SD = 0.77), “Scientists' work improves people's lives” (D.7; *M* = 2.89; SD = 0.88), “Scientific theories are reliable” (D.12; *M* = 2.78; SD = 0.82), and “People who understand science make better health decisions” (D.14; *M* = 2.73; SD = 0.90). Conversely, negatively worded items expressing skepticism toward science received the lowest scores: “Scientific theories are weak explanations” (D.3; *M* = 0.91; SD = 0.80), “When scientists formulate a hypothesis, they are just guessing” (D.13; *M* = 1.05; SD = 0.83), and “Scientists do not value others' ideas” (D.6; *M* = 1.43; SD = 0.82). Complete scores for all 14 items are reported in Table 7.

**Table 7:** Mean scores and standard deviations of the Likert scale for attitudes toward science (*N* = 407; scale 0–4).

Item	Description	Mean	SD
D.1	When scientists change their mind on a concept, I change mine too	1.53	0.93
D.2	Scientists ignore evidence that contradicts their work	1.48	0.86
D.3	Scientific theories are weak explanations	0.91	0.80
D.4	Scientists intentionally keep their work secret	1.60	0.96
D.5	We can trust that scientists share their discoveries	2.12	0.85
D.6	Scientists do not value others' ideas	1.43	0.82
D.7	I trust that scientists' work improves people's lives	2.89	0.88
D.8	Scientists do not care whether ordinary people understand their work	1.91	0.84
D.9	We should trust the work of scientists	2.95	0.77
D.10	We should trust that scientists are honest	2.61	0.80
D.11	We should trust that scientists behave ethically	2.58	0.88
D.12	Scientific theories are reliable	2.78	0.82
D.13	When scientists formulate a hypothesis, they are just guessing	1.05	0.83
D.14	People who better understand science make better health decisions	2.73	0.90

### 3.8 Gender differences in attitudes toward science

Using two-sided independent-samples *t*-tests, no item showed statistically significant mean differences between male and female students at the 0.05 significance level. However, chi-square analyses of response distributions revealed statistically significant sex differences for the following specific items: sharing of scientific discoveries (D.5; *p* = 0.016), trust in scientists' work (D.9; *p* = 0.018), ethics of scientific work (D.11; *p* = 0.003), reliability of scientific theories (D.12; *p* = 0.001), and understanding of science (D.14; *p* < 0.001). Complete results of the sex comparison are reported in Table 7.

### 3.9 Exploratory logistic regression analysis: differential attitudinal patterns by sex

The secondary exploratory stepwise logistic regression analysis identified four attitudinal variables differentially associated with female sex. As noted in the Methods, the model was used for group discrimination rather than causal or explanatory inference. The odds ratios reported below should therefore be interpreted strictly as descriptive measures of differential association between male and female participants and not as evidence of prediction, causation, or determination of biological sex:

- Ethics of scientific work (D.11): OR = 1.61 (95% CI: 1.13–2.31; *p* = 0.009), positive association with female gender.
- Agreement that scientific theories are weak explanations (D.3): OR = 1.38 (95% CI: 1.00–1.89; *p* = 0.047), positive association with female gender. Given the negatively worded phrasing of this item, the direction of this association requires contextualization
- Perceived reliability of individuals as sources (C.2): OR = 0.689 (95% CI: 0.56–0.85; *p* = 0.001), negative association with female gender.
- Perception of scientific influence (C.3): OR = 0.745 (95% CI: 0.57–0.97; *p* = 0.032), negative association with female gender.

The variable understanding of science (D.14) did not reach statistical significance in the final model (OR = 0.768; *p* = 0.067) and was therefore not retained in the final model as a significant discriminating variable The overall model reached statistical significance [LR chi^2^ (7) = 29.70, *p* < 0.001], formal goodness-of-fit assessment (Hosmer-Lemeshow test) was not conducted. Given the conceptual limitations of this modeling approach, all results are interpreted as exploratory observations only.

### 3.10 Analysis by year of study

To describe the distribution of main study variables, a descriptive analysis stratified by year of study was performed. Given the marked concentration of participants in the first year (*n* = 291; 71.50%) and the small sample sizes in subsequent years, formal statistical comparisons between groups were not performed. The results of the descriptive stratified analysis are presented in Table 8.

**Table 8:** Main study variables stratified by year of study (*N* = 407).

Variable	1st year (*n* = 291)	2nd year (*n* = 14)	3rd year (*n* = 15)	4th year (*n* = 36)	5th year (*n* = 16)	6th year (*n* = 28)	Out (*n* = 7)
Use of homeopathy (%)	21.31	21.43	20.00	25.00	12.50	17.86	28.57
Recommends alternatives (%)	10.65	0.00	13.33	2.78	6.25	3.57	0.00
Physician most reliable (%)	93.47	100.00	93.33	94.44	100.00	100.00	100.00
D.9 agree/strongly agree (%)	71.13	85.71	80.00	88.89	87.50	100.00	85.71
D.7 agree/strongly agree (%)	65.29	78.57	73.33	86.11	93.75	100.00	85.71
D.1 agree/strongly agree (%)	73.88	85.71	80.00	86.11	87.50	96.43	85.71

Use of homeopathy by year of study. The prevalence of homeopathic product use remained substantially stable across years of study, with values ranging from 12.50% in the fifth year to 25.00% in the fourth year. First-year students demonstrated a prevalence of 21.31%, consistent with the overall sample prevalence (21.13%).

Propensity to recommend alternative methods by year of study. The propensity to recommend alternative remedies was lower in more advances years of study. First-year students demonstrated the highest prevalence (10.65%), while from the second year onwards a marked reduction was observed: 100% of second-year students reported not recommending alternative remedies, and markedly reduced percentages were recorded in the fourth year (2.78%), fifth year (6.25%), and sixth year (3.57%). Perceived reliability of the physician by year of study. Trust in the physician remained high; cross-sectionally, the proportion identifying the physicianas the most reliable source was higher in moreadvanced cohorts: in the first year, 93.47% of students indicated the physician as the most reliable source, a percentage that was 100.00% in the second, fifth, and sixth years and among out-of-programme students.Attitudes toward science by year of study, key items. The descriptive analysis revealed higher observed frequencies of positive attitudes toward science in more advanced years of study. For item D.9 “Trust in scientists' work,” the percentage of “agree” or “strongly agree” responses was higher in more advanced cohorts: 71.13% in the first year, 85.71% in the second year, 80.00% in the third year, 88.89% in the fourth year, 87.50% in the fifth year, and 100.00% in the sixth year. A similar trend was observed for item D.7 (65.29% in the first year → 100.00% in the sixth year) and for item D.1 (73.88% in the first year; 96.43% in the sixth year).

## 4 Discussion

The findings of this study offer a multidimensional picture of how university students relate to health information in the digital environment, revealing both encouraging attitudinal foundations and gaps in self-reported critical evaluation abilities, that carry direct implications for educational policy and practice. The sociodemographic profile of the sample is consistent with the broader academic landscape of health-related faculties in southern Italian universities. The predominance of students enrolled in Nursing (65.36%) and Medicine and Surgery (23.34%) reflects the institutional composition of the University Magna Graecia of Catanzaro (UMG), where these two programmes attract the largest student populations (42, 43). The strong concentration of participants in the first year (71.50%) is a natural consequence of the convenience sampling strategy adopted, which involved students attending scheduled classroom activities, a recruitment context that tends to favor higher participation rates among early-year cohorts (44). These compositional features reflect the structural characteristics of the academic context investigated: the findings therefore offer a detailed portrait of the attitudes and digital health literacy profiles of first-year Nursing students at a southern Italian university, a population that occupies a central position in the educational pipeline for healthcare professionals.

The marked predominance of female students (68.06%) is consistent with well-established national and international trends in health-related degree programmes, particularly Nursing, where a gender imbalance in favor of women has been consistently documented across diverse academic contexts (45, 46). Notably, the sample also included students from non-health disciplines, such as Engineering (2.46%), reflecting the multidisciplinary character of UMG and suggesting that digital health literacy may carryrelevance beyond health-focused curricula;however, the minimal representation of non-health students (Engineering: *n* = 10, 2.46%) limits the generalisability of this observation. The finding that three quarters of students (75.43%) rely on social media as their primary health information source is noteworthy, yet consistent with the existing literature (7, 19, 47). Of particular concern is the juxtaposition of this finding with the low prevalence of self-reported advanced critical evaluation abilities: only 15.72% of students reported being able to critically appraise the information they retrieve online, suggesting that the vast majority perceive themselves as lacking adequate methodological tools (48, 49). It is important to acknowledge that this figure reflects students' self-reported perception of their own competence, rather than performance derived from objective assessment. The discrepancy between perceived and actual proficiency in health information evaluation is a well-documented phenomenon in the health literacy literature, whereby individuals systematically overestimate their critical appraisal abilities when evaluated against standardized measures (50, 51). More broadly, this caveat applies to the entirety of the digital health literacy data reported in this study. All competency-related findings reflect students' self-assessment of their own abilities and should not be interpreted as measures of actual performance. The systematic overestimation of information evaluation skills documented in the literature suggests that the true prevalence of adequate critical competencies in this population may be lower than the self-reported figures indicate (51). Vrdelja et al. (52), in a systematic review on health information-seeking behavior among university students, demonstrated that social media use for health-related purposes is consistently associated with greater exposure to misinformation and a reduced tendency to cross-verify information through authoritative sources. Similarly, Sundell et al. (47) documented that while digital health information-seeking was highly prevalent, health literacy levels were markedly heterogeneous, with a substantial proportion of students lacking the critical competencies required to evaluate online content systematically. Taken together, these findings converge on a shared conclusion: uncritical reliance on social media for health information is a structural feature of contemporary student populations that warrants a coordinated and systematic educational response (53, 54). Dadaczynski et al. (55) demonstrated that higher levels of digital health literacy are significantly associated with better ability to identify misinformation and with more protective health behaviors during the COVID-19 pandemic, suggesting that investing in the development of advanced critical competencies could yield measurable Public Health benefits. When students do attempt to evaluate the quality of health information, the criteria they apply tend to reflect surface-level heuristics rather than rigorous methodological thinking. The most frequently cited criteria, up-to-date content (49.14%) and information provided by health professionals (45.21%), are certainly relevant, but they remain insufficient as standalone indicators of information quality. More concerning is the markedly reduced prevalence of criteria reflecting deeper methodological reasoning: cross-checking with multiple websites was cited by only 26.29% of participants, consultation with a health professional by 16.46%, and transparency of intentions/purposes by only 14.50%, the least frequently adopted criterion overall. This reliance on cognitive shortcuts aligns closely with what Flanagin et al. (3) described as heuristic credibility assessment: a mode of information appraisal characterized by rapid judgements based on perceived source reputation and surface-level cues, rather than systematic and structured verification. The contrast with evidence-based evaluation strategies is stark. The prevalence of homeopathic product use (21.13%) and the propensity to recommend alternative remedies (9.09%) observed in this sample are consistent with data reported in the international literature on health students. Jocham et al. (56) documented that approximately one in five medical students reported personal use of homeopathic products, with a higher prevalence among female students, a finding consistent with the sex difference observed in the present sample (females 23.10% vs. males 16.92%). Hall et al. (57) documented widespread favorable attitudes toward complementary therapies even among healthcare professionals, while Samara et al. (58) highlighted that personal use is associated with professional recommendation, suggesting a moderating role of evidence-based training. This is consistent with the hypothesis that clinical and methodological training may orient students toward an evidence-based approach; however, this interpretation cannot be substantiated on the basis of cross-sectional data alone (59). One of the most striking and reassuring findings of the present study is the near-unanimous identification of the physician as the most reliable health information source, endorsed by 94.10% of participants. This finding stands in apparent contrast with the dominance of social media as the primary information channel; however, the two observations are not necessarily contradictory. Rather, they may reflect a pragmatic and hierarchically coherent information management strategy: students consult social media for rapid, everyday access to health content while reserving the physician as the ultimate epistemic authority for clinically relevant decisions (60, 61). From an educational standpoint, this finding is encouraging: the strong institutional trust in the physician suggests that students already possess a solid epistemic reference point, which may serve as a foundation upon which more structured approaches to health information evaluation can be developed (60). The attitudinal profile emerging from the Likert scale analysis paints an encouraging picture. Students demonstrated consistently positive orientations toward science, with the highest mean scores recorded for trust in scientists' work (D.9; *M* = 2.95), the beneficial impact of science on daily life (D.7; *M* = 2.89), and the reliability of scientific theories (D.12; *M* = 2.78). Equally notable is the strong rejection of skeptical and conspiratorial propositions: negatively worded items asserting that scientific theories are weak explanations (D.3; *M* = 0.91) or that scientists merely guess their hypotheses (D.13; *M* = 1.05) received the lowest scores in the entire scale, indicating widespread disagreement with anti-scientific narratives. This differentiates university students, particularly those in health and scientific programmes, from segments of the general public where mistrust in science has been more widely documente (62–65). Lee and Ramazan (63) demonstrated that higher media literacy and health literacy are independently associated with a greater capacity for fact-checking health information, suggesting that positive epistemological attitudes and critical competencies may jointly operate as a cognitive protective factor against false health narratives. The absence of statistically significant mean differences between males and females on any of the 14 Likert items (two-sided independent-samples *t*-tests, *p* > 0.05 for all items) is consistent with findings from recent studies on health students, which document a progressive convergence of sex differences in epistemological attitudes with increasing educational level (66, 67). These patterns are broadly consistent with theoretical and empirical frameworks documenting that gender differences in risk perception and trust in scientific and health information are shaped by differential gender socialization and culturally mediated orientations toward science and information sources (68–70). Nevertheless, chi-square analyses of response distributions revealed statistically significant sex differences for specific items: sharing of scientific discoveries, trust in scientists' work, ethics of scientific work, reliability of scientific theories, and understanding of science. These findings indicate that sex differences operate at the level of response distribution rather than at the level of mean scores, and that aggregate comparisons may conceal meaningful gender-specific patterns. The logistic regression model was employed asan exploratory group-discrimination tool,analogous to discriminant function analysis, to identify which attitudinal and behavioral variables most strongly differentiate male from female students. The odds ratios quantify the degree to which each variable discriminates between the two groups and should not be interpreted as implying that the variables in the model causally determine biological sex. While not the conventional epidemiological framing, this approach finds precedent in sex- and gender-score methodology in peer-reviewed health survey research (38, 40). It is acknowledged that these methodological precedents arise primarily from classification and gender-score development contexts, and that the present analysis constitutes a secondary exploratory exercise rather than a primary explanatory model of digital health literacy or attitudes toward science. It should furthermore be noted that the odds ratios obtained are modest in magnitude and that confidence intervals for several variables approach the null.

The descriptive analysis stratified by year of study reveals the following cross-sectional patterns: higher frequencies of pro-scientific attitudes were observed in more advanced student cohorts. The proportion of students expressing trust in scientists' work was 71.13% in the first year and 100.00% in the sixth year, while similar patterns were observed for items related to the beneficial impact of science and the reliability of scientific theories. Concurrently, the propensity to recommend alternative remedies declines substantially across years, from 10.65% among first-year students to just 3.57% among those in the sixth year. It should be noted that, given the marked concentration of participants in the first year (71.50%) and the small sample sizes in subsequent years, these patterns are stricltly descriptive in nature, were not formally tested for statistical significance and cannot be interpreted as evidence of individual developmental change. These cross-sectional observations are consistent with existing literature on the relationship between university education in health programmes and epistemological attitudes toward science (71, 72). Structured educational interventions appear to accelerate this process: Spracklin et al. documented significant improvements in the ability to evaluate online health information following the integration of lateral reading and SIFT strategies into nursing curricula (72). The findings of this study point to a central and actionable insight: the gap between epistemological orientation and self-reported operational competence ability is not a fixed condition, but a precise map of an educational opportunity. These findings gain further interpretive depth when considered in light of the broader literature on self-report bias in digital health literacy research. Measures relying exclusively on self-declared behaviors, such as item B.2 in the present study, assess what respondents believe they can do, rather than what they are able to demonstrate in practice. Empirical evidence consistently indicates that perceived and objectively measured health literacy are empirically distinct constructs: Schulz and colleagues documented only a weak correlation (*r* = 0.06) between perception-based and performance-based health literacy, and concluded that self-rated assessments should not be used as proxies for actual competence (73). Validation research on self-report instruments in this domain has further revealed that respondents' perceived proficiency systematically exceeds their demonstrated performance on task-based items (74, 75). This pattern is consistent with the metacognitive model formulated by Kruger and Dunning (76), who demonstrated that individuals with limited competence in a given domain tend to lack awareness of that very limitation, resulting in systematic self-assessment inflation. Liu et al. provided direct empirical support for this phenomenon in a digital health literacy context: participants reporting adequate self-assessed digital health literacy scores nonetheless frequently misclassified inaccurate online health content, underscoring the gap between perceived and actual information appraisal competence (77). These considerations reinforce the call for future studies to complement self-report instruments with objective performance-based measures, in order to obtain a more accurate and actionable characterization of students' critical information evaluation abilities. Students in this sample hold positive, well-structured attitudes toward science and maintain a clear epistemic hierarchy that places the physician at the apex of reliable sources. Yet these attitudinal assets have not yet been translated into self-reported critical evaluation abilities skills for systematically appraising the health information they encounter daily on social media. Epistemic trust, however solid, does not automatically generate the operational capacity to distinguish reliable from misleading content in a complex and rapidly evolving digital environment (7, 54, 78, 79). Importantly, these critical digital health literacy competencies are learnable and teachable through targeted educational interventions. Almulhem and Aldekhyyel (60) demonstrated that structured workshops on social media health content evaluation effectively develop critical appraisal skills, suggesting that the university environment, particularly the early academic years, represents the optimal window for targeted epistemological interventions. The observed gap between positive scientific attitudes and limited critical evaluation skills is not an educational failure but an addressable deficit, and the cross-sectional pattern of higher attitudinal scores in more advanced cohorts is consistent with the hypothesis that university education provides a favorable foundation. The cross-sectional design of the present study precludes causal inference, and the observed associations should not be interpreted as evidence that enhancing digital health literacy would necessarily improve misinformation resilience; prospective intervention studies are required to establish such a relationship. Moreover, several individual-level factors that were not captured in the present study, including prior educational background, socioeconomic status, previous health training, academic achievement, media exposure, and frequency of social media use, may independently shape digital health literacy and constitute alternative explanations for the patterns observed. Systematically integrating media literacy, lateral reading, and digital source evaluation into university health curricula represents a strategic investment in the epistemic resilience of future health professionals.

### 4.1 Strengths and limitations

This study presents several methodological and scientific strengths that reinforce its validity and relevance in the Italian research landscape on digital health literacy and attitudes toward science among university students in health programmes. The study presents several methodological strengths. The sample of over four hundred students provides sufficient statistical power for all analyses performed. The multidimensional approach, simultaneously exploring information sources, digital health literacy, evaluation criteria, use of alternative remedies, and attitudes toward science, represents an original contribution to the Italian literature, particularly given the focus on health programme students. Finally, the use of a Likert scale informed by established literature (16) allowed quantitative measurement of the epistemological, ethical, and trust-related dimensions of students‘ relationship with science. This study presents some limitations that must be considered when interpreting the results. The first and most significant limitation concerns the use of convenience sampling, which does not allow for generalization of the results to the entire student population of UMG or to Italian university students more broadly. The strong concentration of participants in the first year (71.50%) may have introduced a selection bias in the stratified analyses by year of study, limiting the representativeness of the subgroups of advanced years. The use of a convenience sampling strategy, combined with the reliance on face-to-face teaching sessions as the primary recruitment context, further limits the representativeness of the sample. Although the discipline distribution broadly reflects institutional enrolment patterns, the findings cannot be generalized to the broader Italian university population. Future research should employ probabilistic sampling designs and include multi-site recruitment strategies to strengthen external validity. A second limitation concerns the self-reported nature of the data: the declared competencies of digital health literacy may not accurately reflect students' actual operational abilities, as the literature has documented a systematic tendency to overestimate one's own information evaluation skills. A third limitation concerns the cross-sectional nature of the study design, which does not allow for causal inferences between academic progression and the evolution of attitudes toward science or digital health literacy skills (80, 81). In particular, apparent differences across academic years cannot be interpreted as evidence of developmental change, as cross-sectional designs are fundamentally unable to disentangle cohort effects from within-individual trajectories. A fourth limitation concerns the absence of formal psychometric validation of the data collection instrument. The questionnaire was developed *ad hoc* by the research team, informed by the available scientific literature, and subjected to expert review for content clarity and relevance. However, no internal consistency analysis, exploratory factor analysis, or test-retest reliability assessment was conducted. Consequently, the psychometric properties of the instrument remain undetermined, and the findings derived from its administration should be interpreted with appropriate caution. Future studies should address these limitations through the adoption of probabilistic sampling strategies, the use of validated instruments, and longitudinal designs capable of tracking the evolution of competencies and attitudes over the course of university training. A fifth limitation concerns the management of multiple-response variables (B.1, B.2, B.3): frequencies were computed per response option rather than per respondent, which precludes standard multivariate analysis of these items. Finally, as with all self-reported survey data, the possibility of social desirability bias, recall bias, and measurement error cannot be fully excluded. A further limitation concerns the absence of effect size measures. Statistically significant differences identified in both the univariate analyses and the regression model were not accompanied by estimates of practical magnitude, such as Cramér's V for chi-square associations or Cohen's d for group comparisons. The unequal distribution of participants across academic years, with first-year students representing the large majority of the sample, limits the interpretability of cross-cohort comparisons, which should be read as descriptive observations rather than generalisable findings. Consequently, the practical significance of the observed differences cannot be determined from the current data, and the inclusion of standardized effect size indices is recommended in future studies. A further limitation concerns the absence of correction for multiple comparisons. Given the exploratory purpose of the analyses, no adjustment procedure was applied; however, the large number of statistical tests performed increases the probability of false-positive findings, and the reported *p*-values should be interpreted accordingly. A final concern is the exploratory use of a stepwise logistic regression approach, which may increase the risk of model instability (82, 83). Additionally, standard logistic regression diagnostic procedures were not performed: variance inflation factors (VIF) for the assessment of multicollinearity and the Hosmer-Lemeshow goodness-of-fit test were not applied, which precludes a comprehensive evaluation of model adequacy. Consequently, these findings should be interpreted with caution, and future studies should employ theory-driven variable selection strategies, accompanied by standard diagnostic checks, to improve the robustness and interpretability of the regression models. Concerning the logistic regression model, the use of biological sex as the dependent variable, while documented in sex- and gender-score methodology in peer-reviewed health research (38, 40), represents a non-conventional analytical choice;the exploratory findings should therefore be interpreted with caution and replicated using more standard modeling approaches in future studies.

### 4.2 Implications for practice and research

The findings of this study offer preliminary insights with potential implications for the planning of educational interventions in university contexts. The documented gap between basic digital health literacy skills and advanced critical evaluation skills suggests the need to integrate into university curricula, particularly in health programmes, specific modules dedicated to the critical evaluation of online health information, including the lateral reading approach, fact-checking strategies, and recognition of the main markers of scientific disinformation. The cross-sectional differences observed across years of study are consistent with thehypothesis that the curriculum may exert a positive socializing effect on epistemologicalorientations, but that this effect needs to be made more explicit and systematic throughtargeted educational interventions. The differential associations observed between selected attitudinal variables and biological sex may help generate hypotheses for future research on sex-related patterns in digital health literacy and attitudes toward science. Given the exploratory and group-discrimination nature of the regression analysis, these findings should not at this stage be used to infer causal mechanisms or to justify sex-specific educational interventions. From a research perspective, this study highlights the need for longitudinal studies capable of tracking the evolution of digital health literacy skills and attitudes toward science over the course of university training, as well as for intervention studies capable of evaluating the effectiveness of specific educational programmes for the development of critical evaluation skills in the health domain.

## 5 Conclusions

This study documents a structural tension in the relationship of UMG health students with health information: solid epistemological trust in science and physicians coexists with self-reported digital health literacy abilities still in development, particularly regarding critical evaluation and conscious content sharing. The attitudinal foundations observed in this sample are consistent with an orientation toward scientific culture, yet the translation of these orientations into operational competencies is not automatic and may benefit from targeted educational support. These findings, while limited to the population studied, support the integration of media literacy, lateral reading, and digital source evaluation into health degree curricula as a strategic priority for preparing students to navigate the infodemic era with methodological awareness.

## Data Availability

The raw data supporting the conclusions of this article will be made available by the authors, without undue reservation.
