# Supplementary material for: Health misinformation, digital health literacy, and attitudes toward science among university students: an exploratory cross-sectional survey
Source: Front Public Health. 2026 Aug 31;14:1931903. doi: 10.3389/fpubh.2026.1931903 (PMC13572223; doi:10.3389/fpubh.2026.1931903)
Supplement: Supplementary file 1 [file Data_Sheet_1.pdf]

Supplementary Material 1

Survey Instrument

Fake News and Public Health

Note: The questionnaire was administered in Italian to health science students at the University Magna Graecia of Catanzaro (UMG), Italy. The instrument is presented below in its original language as administered to participants.

Missing data code: 99 | Not applicable code: 88

Section A — Sociodemographic Characteristics

A1. Qual è la tua età in anni compiuti? [Age in completed years]

_______ years

A2. Qual è il tuo genere? [What is your gender?]

- ◻ Maschile [Male]
- ◻ Femminile [Female]
- ◻ Non binario [Non-binary]

A3. Qual è il tuo stato civile? [What is your marital status?]

- ◻ Nubile/Celibe [Single]
- ◻ Sposata/o – Convivente [Married/Cohabiting]
- ◻ Separata/o – Divorziata/o [Separated/Divorced]
- ◻ Vedova/o [Widowed]

A4. In quale ateneo studia? [At which university do you study?]

- ◻ UMG
- ◻ UNICAL
- ◻ Altro [Other]

A5. Qual è il corso di studi da lei frequentato? [Which degree programme are you enrolled in?]

- ◻ Medicina e Chirurgia [Medicine and Surgery]
- ◻ Ingegneria [Engineering]
- ◻ Scienze Infermieristiche [Nursing Sciences]

- □ Altro [Other]

A6. A quale anno di corso è iscritto? [Which year of study are you in?]

- □ Fuori Corso [Out of course]
- □ 1°
- □ 2°
- □ 3°
- □ 4°
- □ 5°
- □ 6°

Section B — Health Information Access and Quality

B1. Da chi, solitamente, riceve/ascolta informazioni riguardo la salute pubblica/scienza? (sono possibili più risposte) [From whom do you usually receive health/science information? Multiple answers allowed]

- □ Social [Social media]
- □ Medico di Famiglia [General Practitioner]
- □ Televisione [Television]
- □ Amici e Conoscenti [Friends and Acquaintances]
- □ Non è di mio interesse [Not of interest to me]

B2. Qual è la sua esperienza e capacità di accesso a informazioni relative alla salute? (sono possibili più risposte) [What is your experience and ability in accessing health information? Multiple answers allowed]

- □ Quando incontro informazioni sulla salute, navigo su Internet per ulteriori informazioni correlate [When I encounter health information, I browse the Internet for related information]
- □ Quando voglio ottenere informazioni sulla salute, generalmente navigo sui siti web di istituzioni pubbliche sanitarie o ospedali [I generally browse public health institution or hospital websites]
- □ Ho esperienza nel trovare informazioni relative alla salute su Internet [I am experienced in finding health information online]
- □ So dove cercare informazioni sulla salute su Internet [I know where to look for health information online]

- □ So quali termini di ricerca usare per trovare informazioni relative alla salute su Internet [I know which search terms to use]
- □ Posso scegliere le informazioni desiderate dal vasto insieme di informazioni disponibili su Internet [I can select the desired information from the large amount available online]

B3. Come valuta la qualità dell'informazione relativa alla salute? (sono possibili più risposte) [How do you assess the quality of health information? Multiple answers allowed]

- □ Considero se le informazioni sulla salute che trovo su Internet siano accurate [I consider accuracy]
- □ Verifico se le informazioni siano aggiornate [I check whether information is up to date]
- □ Controllo le fonti delle informazioni [I check the sources]
- □ Verifico se le informazioni siano fornite da professionisti sanitari [I check if provided by health professionals]
- □ Controllo se le informazioni siano corroborate da evidenze scientifiche [I check scientific evidence]
- □ Controllo eventuali intenzioni o scopi dietro le informazioni [I look for hidden intentions or purposes]
- □ Controllo se le informazioni evidenziano solo gli effetti positivi o nascondono i rischi [I check for one-sided framing]
- □ Confronto le informazioni su più siti web [I cross-reference across multiple websites]
- □ Consulto un professionista sanitario per verificare le informazioni [I consult a health professional]

B4. Ha mai fatto uso di omeopatici? [Have you ever used homeopathic products?]

- □ Sì [Yes]
- □ No [No]

Section C — Attitudes toward Science and Health Information

C1. Riguardo le seguenti dichiarazioni, con quale sei più in accordo? (sono possibili più risposte) [Which of the following statements do you most agree with? Multiple answers allowed]

- □ Gli scienziati/medici dovrebbero parlare della scienza, senza esporre il loro pensiero [Scientists/physicians should speak about science without expressing personal opinions]
- □ Gli scienziati/medici dovrebbero stare al di fuori della politica [Scientists/physicians should stay out of politics]
- □ Gli scienziati/medici sanno qual è la cosa migliore per la salute pubblica [Scientists/physicians know what is best for public health]
- □ Gli scienziati/medici dovrebbero porre più attenzione ai desideri dei cittadini [Scientists/physicians should pay more attention to citizens' preferences]
- □ Altro [Other]

C2. Per quanto riguarda l'informazione nella salute pubblica, chi metteresti al primo posto? [Regarding public health information, who would you trust most?]

- □ Medico [Physician]
- □ Politico [Politician]
- □ Autorità Religiosa [Religious Authority]
- □ Gruppo di Cittadini [Citizens' Group]
- □ Amici e Famiglia [Friends and Family]
- □ Giornalisti [Journalists]
- □ Organizzazioni non governative [NGOs]

C3. Qual è la probabilità che l'ambiente scientifico sia in qualche modo influenzato/manipolato da enti terzi? [How likely is it that the scientific community is influenced/manipulated by external parties?]

- □ Certa [Certain]
- □ Possibile [Possible]
- □ Probabile [Probable]
- □ Improbabile [Unlikely]
- □ Impossibile [Impossible]

C4. Consiglierebbe ad un suo caro di ricorrere a cure/rimedi 'alternativi' non basati sulle evidenze scientifiche? [Would you recommend non-evidence-based alternative treatments to a loved one?]

- □ Sì [Yes]
- □ No [No]

Section D — Trust in Science and Scientists

For each statement, indicate your level of agreement: 0 = Fortemente in disaccordo [Strongly disagree] · 1 = In disaccordo [Disagree] · 2 = Non mi esprimo/neutrale [Neutral] · 3 = In accordo [Agree] · 4 = Fortemente in accordo [Strongly agree]

D1. Quando gli scienziati cambiano idea su un concetto scientifico, questo diminuisce la mia fiducia nel loro lavoro. [When scientists change their mind about a scientific concept, this decreases my trust in their work.]

- □ 0 – Strongly disagree
- □ 1 – Disagree
- □ 2 – Neutral
- □ 3 – Agree
- □ 4 – Strongly agree

D2. Gli scienziati ignorano le prove che contraddicono il loro lavoro. [Scientists ignore evidence that contradicts their work.]

- □ 0 – Strongly disagree
- □ 1 – Disagree
- □ 2 – Neutral
- □ 3 – Agree
- □ 4 – Strongly agree

D3. Le teorie scientifiche sono spiegazioni deboli. [Scientific theories are weak explanations.]

- □ 0 – Strongly disagree
- □ 1 – Disagree

- □ 2 – Neutral
- □ 3 – Agree
- □ 4 – Strongly agree

D4. Gli scienziati tengono intenzionalmente segreto il loro lavoro. [Scientists intentionally keep their work secret.]

- □ 0 – Strongly disagree
- □ 1 – Disagree
- □ 2 – Neutral
- □ 3 – Agree
- □ 4 – Strongly agree

D5. Possiamo fidarci che gli scienziati condividano le loro scoperte anche se non apprezzano i risultati. [We can trust scientists to share their findings even if they dislike the results.]

- □ 0 – Strongly disagree
- □ 1 – Disagree
- □ 2 – Neutral
- □ 3 – Agree
- □ 4 – Strongly agree

D6. Gli scienziati non danno valore alle idee degli altri. [Scientists do not value other people's ideas.]

- □ 0 – Strongly disagree
- □ 1 – Disagree
- □ 2 – Neutral
- □ 3 – Agree
- □ 4 – Strongly agree

D7. Mi fido del fatto che il lavoro degli scienziati migliori la vita delle persone. [I trust that scientists' work improves people's lives.]

- ☐ 0 – Strongly disagree
- ☐ 1 – Disagree
- ☐ 2 – Neutral
- ☐ 3 – Agree
- ☐ 4 – Strongly agree

D8. Gli scienziati non si preoccupano se le persone comuni comprendono il loro lavoro. [Scientists do not care whether ordinary people understand their work.]

- ☐ 0 – Strongly disagree
- ☐ 1 – Disagree
- ☐ 2 – Neutral
- ☐ 3 – Agree
- ☐ 4 – Strongly agree

D9. Dovremmo fidarci del lavoro degli scienziati. [We should trust the work of scientists.]

- ☐ 0 – Strongly disagree
- ☐ 1 – Disagree
- ☐ 2 – Neutral
- ☐ 3 – Agree
- ☐ 4 – Strongly agree

D10. Dovremmo fidarci che gli scienziati siano onesti nel loro lavoro. [We should trust scientists to be honest in their work.]

- ☐ 0 – Strongly disagree
- ☐ 1 – Disagree
- ☐ 2 – Neutral
- ☐ 3 – Agree
- ☐ 4 – Strongly agree

D11. Dovremmo fidarci che gli scienziati si comportino in modo etico nel loro lavoro. [We should trust scientists to behave ethically in their work.]

- □ 0 – Strongly disagree
- □ 1 – Disagree
- □ 2 – Neutral
- □ 3 – Agree
- □ 4 – Strongly agree

D12. Le teorie scientifiche sono affidabili. [Scientific theories are reliable.]

- □ 0 – Strongly disagree
- □ 1 – Disagree
- □ 2 – Neutral
- □ 3 – Agree
- □ 4 – Strongly agree

D13. Quando gli scienziati formulano un'ipotesi, stanno solo indovinando. [When scientists form a hypothesis, they are just guessing.]

- □ 0 – Strongly disagree
- □ 1 – Disagree
- □ 2 – Neutral
- □ 3 – Agree
- □ 4 – Strongly agree

D14. Le persone che comprendono meglio la scienza hanno maggiore fiducia nella scienza. [People who better understand science have greater trust in science.]

- □ 0 – Strongly disagree
- □ 1 – Disagree
- □ 2 – Neutral
- □ 3 – Agree
- □ 4 – Strongly agree
